# Case Report: Reemerging Paragonimiasis in Umphang District, Thailand

**DOI:** 10.4269/ajtmh.22-0708

**Published:** 2023-02-27

**Authors:** Jiranat Hanprom, Keswadee Lapphra, Worawit Tontiwattanasap, Ratchadaporn Papwijitsil, Katherine Copeland, Kulkanya Chokephaibulkit

**Affiliations:** ^1^ Umphang Hospital, Tak, Thailand;; ^2^ Department of Paediatrics, Faculty of Medicine Siriraj Hospital, Mahidol University, Bangkok, Thailand;; ^3^ Division of Epidemiology, Department of Disease Control, Ministry of Public Health, Nonthaburi, Thailand;; ^4^ Department of Sciences, Mahidol University International College, Nakhon Pathom, Thailand;; ^5^ Siriraj Institute of Clinical Research, Mahidol University, Thailand

## Abstract

Paragonimiasis is a food-born zoonotic parasitosis caused by *Paragonimus* spp. Six cases of reemerging paragonimiasis within the Karan hill-tribe near the Thai–Myanmar border were evaluated to review clinical manifestations, predisposing factors, and treatment regimens. All patients tested positive for paragonimiasis eggs and presented with an array of symptoms, including chronic cough, hemoptysis, peripheral eosinophilia, and thoracic radiograph abnormalities. All fully recovered after a 2- to 5-day course of 75 to 80 mg/kg/day praziquantel. We conclude that paragonimiasis should be considered during differential diagnoses to promote early treatment and to prevent misdiagnosis of reemerging or sporadic cases. This applies particularly to endemic regions and high-risk groups known to habitually consume raw or undercooked intermediate or paratenic hosts.

## INTRODUCTION

More than 293.8 million people worldwide are at risk of contracting paragonimiasis,^1^^,^^2^ a food-born zoonotic parasitosis of the tropics and subtropics caused by *Paragonimus* spp.^3^^–^^7^ Both infective metacercarial stages of *Paragonimus westermani* and *Paragonimus heterotremus* are endemic to Thailand, the latter being the main reemerging etiological agent in humans.^3^^–^^5^^,^^8^^,^^9^ Infection occurs through consumption of raw, pickled, salted, or cured crustaceans (secondary intermediate hosts) or mammals (paratenic hosts) that harbor live metacercaria.^1^^,^^3^^,^^4^^,^^9^^–^^14^ Paragonimiasis presents initially as an acute febrile illness (e.g., fever, malaise, weight-loss, coughing) that progresses into pleuropulmonary morbidities (e.g., hemoptysis, pleural effusion, pneumothorax, peripheral eosinophilia), particularly of the lower respiratory tract. Extrapulmonary paragonimiasis develops when these immature flukes migrate to ectopic sites, including the central nervous system, pericardium, bone marrow, abdominal organs, reproductive organs, and more.^1^^,^^2^^,^^15^^,^^16^ Sputum, stool, or biopsies can determine the presence of *Paragonimus* eggs and distinguish paragonimiasis from other pulmonary diseases of similar presentation (e.g., tuberculosis [TB], cancer, pneumonia, bronchitis, or parapneumonic effusion).^4^^,^^5^^,^^13^^,^^17^

As paragonimiasis remains a neglected tropical disease according to the WHO,^18^ we sought to review the clinical manifestations, predisposing factors, and treatment of six cases identified in 2017 within the Karan hill-tribe near the Thai–Myanmar border in Umphang District, Tak Province, western Thailand.

## RESULTS

### Case 1.

**Figure 1.**
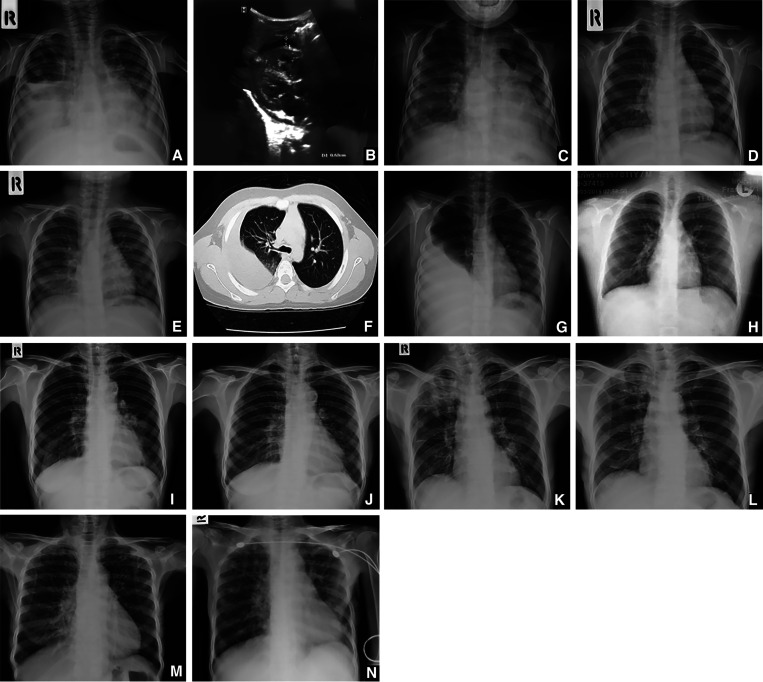
(**A–C**) Thoracic radiographs and ultrasound of a 3-year-old Karan boy. (**A**) The radiograph taken before treatment, with haziness in the right middle lobe (RML) and minimal reticulonodular infiltration in both upper lung fields. (**B**) The ultrasound, with pericardial effusion 0.68 cm in thickness. (**C**) The patient’s radiograph 2 weeks after treatment, with markedly decreased pulmonary infiltration. (**D** and **E**) Thoracic radiographs of a 4-year-old Karan girl. (**D**) The radiograph was taken during initial presentation, with reticular infiltration observed in the RML. (**E**) The effects of treatment after 2 months, with resolution of RML infiltration. (**F–H**) Thoracic radiographs and computed tomographic (CT) scans for a 10-year-old boy. (**F**) The CT scan shows hyperdense pleural effusion, with some calcification in the patient’s right lung. The patient’s radiographs before (**G**) and after (**H**) treatment illustrate decreased pleural effusion after 7 months. (**I** and **J**) Thoracic radiographs of a 55-year-old woman before and after treatment with praziquantel. (**I**) The radiograph during the hospital visit, with an oval ring-shadow with speckled calcification and nodular infiltration observed in the left hilar region (white arrow). There was no evidence of pleural effusion. (**J**) The effects of treatment 2 months later, with decreased infiltration within the left hilar region. (**K** and **L**) Thoracic radiographs of a 60-year-old man. (**K**) The radiograph taken during initial presentation, with nodular infiltration in the right upper lobe and lower left lobe. (**L**) The effects of treatment after 1 month, with partial resolution of pulmonary infiltration. (**M** and **N**) Thoracic radiographs of a 66-year-old woman. (**M**) The radiograph taken during initial presentation, with reticular infiltration in the RML. (**N**) The effects of treatment after 2 months, with faded infiltration.

A 3-year-old Karan boy was severely malnourished, anemic, and suffered from progressive dyspnea and abdominal distension after continually consuming mountain crabs. He had no history of contact TB; sputum, stool, and pleural fluid tested positive for *Paragonimus* eggs. The thoracic radiograph taken during initial onset (Figure 1A) revealed haziness and minimal reticulonodular infiltration in the right middle lobe (RML). Abdominal ultrasound (Figure 1B) confirmed marked ascites, hepatosplenomegaly, and pericardial effusion (0.68 cm). Decreased abdominal distension, pulmonary infiltration, and increased weight (4 kg) were observed (Figure 1C) 14 days after hospitalization and treatment (Table 1).

**Table 1:** Characteristics of six patients diagnosed with paragonimiasis in 2017 in Umphang District, Tak Province, Thailand

Patient characteristics	Clinical presentation, history, and physical findings	Exposure	Laboratory findings	Radiological findings	Treatment
3-Year-old Karan boy, Myanmese village opposite to Mae Chan subdistrict	Nonproductive cough of 1 month; progressive abdominal distention and low-grade fever of 2 weeks; weight, 10 kg (< third percentile); presented with mild tachycardia, tachypnea, fine crepitation in RUL, decreased breath sounds in RLL, SpO_2_ of 94%, distant heart sounds, pale conjunctiva, hepatosplenomegaly, and multiple cervical lymphadenopathies	Consumed undercooked mountain crabs	Hct, 27%; WBC count, 22,510 cells/mm^3^ (55% neutrophils, 31% lymphocytes, 14% eosinophils); platelets, 917,000 cells/mm^3^; total bilirubin, 0.3 mg/dL; aspartate aminotransferase, 54 U/L; alanine transaminase, 55 U/L; globulin, 8.5 g/dL; albumin, 3.3 g/dL; sputum AFB negative; tuberculin skin test negative; stool positive for *Paragonimus* eggs	RML haziness, minimal reticulonodular infiltration in upper lung fields	PZQ, 75 mg/kg/day, thrice daily for 2 days; right intercostal chest drain for 7 days
4-Year-old Karan girl, Mae Chan subdistrict	Chronic cough of 1 year; hemoptysis of 3 days; weight, 12 kg (< fifth percentile); presented with tachypnea, tachycardia, fine crepitation in RLL, and multiple cervical lymphadenopathies	Consumed undercooked mountain crabs	Hct, 31%; WBC count; 9,150 cells/mm^3^ (37% neutrophils, 48% lymphocytes, 12% eosinophils); platelets, 282,000 cell/mm^3^; sputum AFB negative, *Paragonimus* egg positive	RML reticulonodular infiltration	PZQ, 75 mg/kg/day, thrice daily, for 2 days
10-Year-old Karan boy, Mae Klong subdistrict	Persistent pleural effusion of 2 months; cough and progressive dyspnea of 2 weeks; weight, 33 kg (< fifth percentile); presented with mild tachypnea and decreased breath sounds in right lung	No history of consuming raw or undercooked mountain crab	Hct, 39%; WBC count, 10,360 cells/mm^3^ (45% neutrophils, 38% lymphocytes, 5% eosinophils); platelets, 392,000 cells/mm^3^; sputum AFB negative, *Paragonimus* egg positive; tuberculin skin test negative; no granulomas or malignancies from pleural biopsy	RLL haziness, hyperdense pleural effusion with some calcification (14.0 × 10.0 × 16.7 cm)	PZQ, 80 mg/kg/day, thrice daily for 5 days; pigtail catheter drain for 7 days
55-Year-old Karan female farmer, Mae Chan subdistrict	Weight loss of 2 months (> 5 kg; body mass index, 17.7), fatigue and myalgia of 1 month, productive hemoptysis of 2 weeks	No history of consuming raw or undercooked mountain crab	Sputum AFB negative, *Paragonimus* egg positive	Left hilar region nodular infiltration	PZQ, 75 mg/kg/day, thrice daily for 2 days
60-Year-old Karan male farmer, Mae Klong subdistrict	Chronic cough and fatigue of 5 months; progressive, productive cough with brown-tinged sputum of 4 days; weight, 49 kg; presented with cachexia, tachypnea, respiratory distress, and fine crepitation in RUL	No history of consuming raw or undercooked mountain crab	Sputum AFB negative, *Paragonimus* egg positive	RUL reticulonodular infiltration, left lower lobe nodular infiltration	PZQ, 75 mg/kg/day, thrice daily for 2 days
66-Year-old Karan female farmer, Mo Kro subdistrict	Productive cough with hemoptysis, fatigue, and weight loss (> 3 kg) of 1 month; acute diarrhea, cachexia (weight, 33 kg), tachypnea, respiratory distress, coarse precipitation right lung, hemiplegia (muscle power grade IV); neurocysticercosis 3 months prior to presentation	No history of consuming raw or undercooked mountain crab	Hct, 29%; WBC count, 10,160 cells/mm^3^ (19% neutrophils, 24% lymphocytes, 55% eosinophils); platelets, 173,000 cells/mm^3^; sputum AFB negative, *Paragonimus* egg positive	RML reticular infiltration	PZQ, 75 mg/kg/day, thrice daily for 2 days

AFB = acid-fast bacilli; Hct = hematocrit; PZQ = praziquantel; RLL = right lower lobe; RML = right middle lobe; RUL = right upper lobe; SpO_2_ = oxygen saturation; WBC = white blood cell.

### Case 2.

A 4-year-old Karan girl had tachycardia, tachypnea, and fine crepitation in her right lower lobe (RLL) after consuming mountain crabs from the river near her parents’ rice fields. No prior history of fever, chest pain, or contact TB was reported, and sputum test was positive for *Paragonimus* eggs. Figure 1D illustrates her thoracic radiograph during initial onset, with reticulonodular infiltration in the RML. Figure 1E illustrates resolution of this infiltration 2 months after treatment (Table 1).

### Case 3.

A 10-year-old Karan boy presented with cough and progressive dyspnea. Three months earlier, he had dengue hemorrhagic fever. He denied contact TB and his parents denied habitual eating of raw food. A sputum sample tested positive for *Paragonimus* eggs. Figure 1F and G illustrates the presence of hyperdense pleural effusion, with some calcification and haziness of the RLL, respectively. Figure 1H shows the patient’s thoracic radiograph after treatment (Table 1) during a 7-month follow-up, with decreased pleural effusion.

### Case 4.

A 55-year-old Karan woman had productive hemoptysis, fatigue, myalgia, and weight loss. She had no history of fever, chest pain, or contact TB. She did not consume raw mountain crabs, but did drink alcohol habitually. Her sputum sample was positive for *Paragonimus* eggs. Supplemental Figure 1 and Figure 1I illustrate her thoracic radiographs a year prior to admission and during the hospital visit, respectively, with visible nodular infiltration in the left hilar region. Figure 1J shows her thoracic radiograph after a 2-month follow-up, with decreased pulmonary infiltration within the left hilar region and no reported clinical symptoms.

### Case 5.

A 60-year-old Karan man had progressive productive coughing with brown-tinged sputum, and fine crepitation in the right upper lobe (RUL). He drank and smoked actively, but denied weight loss, contact TB, or consuming raw food habitually. His sputum smear was positive for *Paragonimus* eggs. A thoracic radiograph (Figure 1K) revealed multiple reticulonodular and nodular infiltration in the RUL and left lower lobe, respectively. The patient refused further investigation to diagnose paragonimiasis from lung malignancy. A 2-month follow-up revealed decreased pulmonary infiltration in the RUL after treatment (Figure 1L).

### Case 6.

A 66-year-old Karan woman had a productive cough with hemoptysis, cachexia, and coarse precipitation in her right lung. She received treatment of her neurocysticercosis, diagnosed 3 months prior by brain computed tomography, and was on Dilantin for her seizures. She denied history of fever, contact TB, or raw food consumption. Her sputum smear was positive for *Paragonimus* eggs. Figure 1M demonstrates her chest radiograph, with reticular infiltration in the RML. After treatment, this infiltration receded (Figure 1N).

**Figure 2.**
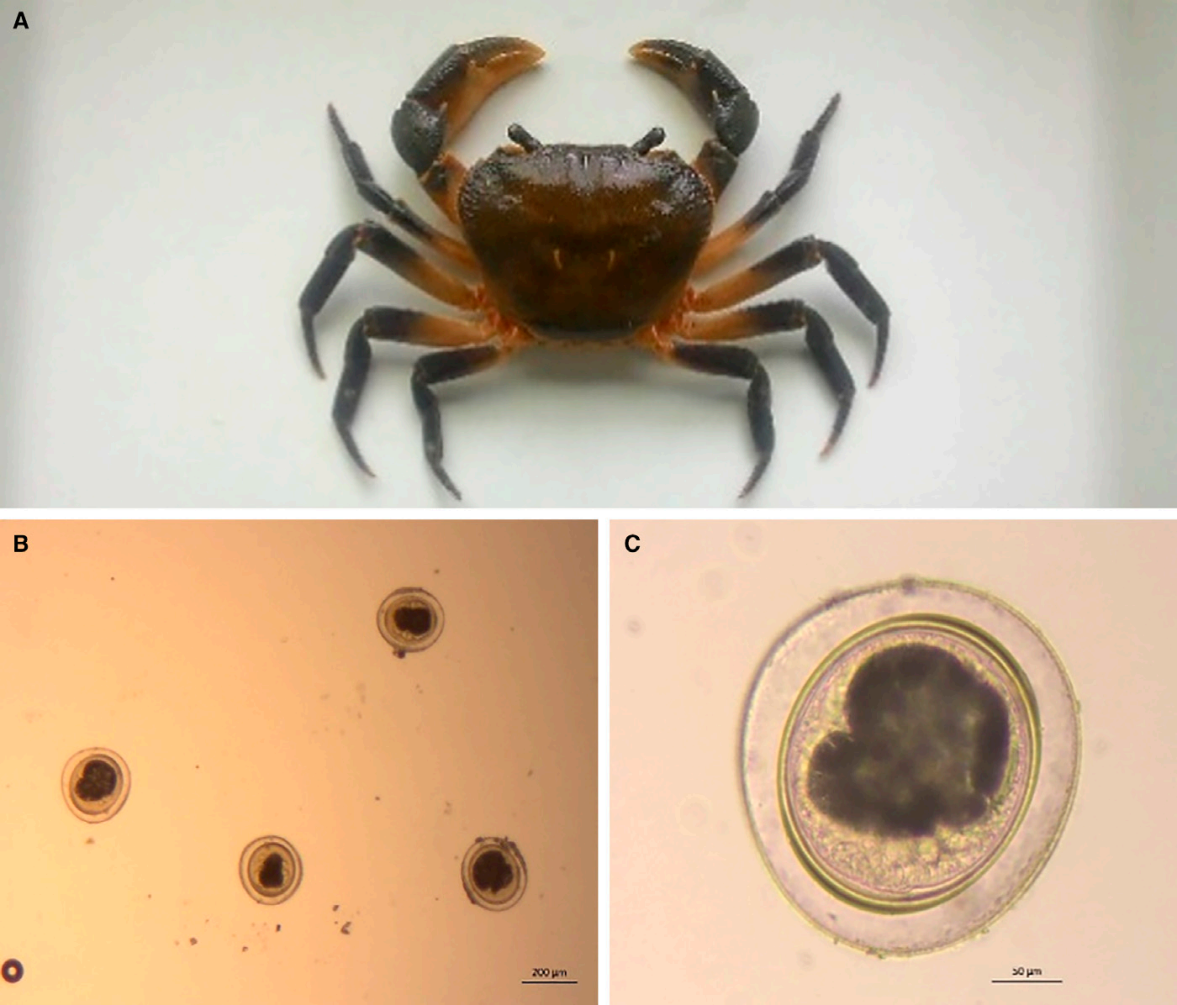
(**A**) One of seven fresh water mountain crabs (14 cm across) retrieved from around Loy Tong Ku’s waterfall, Umphang District, Tak Province, in December 2017. (**B**) The metacercarial phase of *Paragonimus* spp. under a stereomicroscope (×40 magnification). (**C**) Metacercaria (×40 magnification) of the *Paragonimus heterotremus* species complex (includes *P. heterotremus* and *Paragonimus pseudoheterotremus*).

Seven fresh-water mountain crabs (Figure 2A) were collected around Lae-Tong-Ku waterfall in Myanmar. Metacercaria (Figure 2B and C) were identified using a stereomicroscope (×40 magnification) after blending, and polymerase chain reaction confirmed their identities to be *P. heterotremus* and *Paragonimus pseudoheterotremus.* This, coupled with the sudden number of cases, prompted us to arrange health education sessions with patients, community leaders, and villagers to prevent reinfection and future outbreaks.

## DISCUSSION

The incidence of paragonimiasis in Thailand has decreased during the past few decades. Yoonuan et al.^4^ reported that 6.3% of villagers’ sputum samples in Phitsanulok Province tested positive for *Paragonimus* eggs in the 1980 s, and none in 2005. However, the prevalence of metacercaria in intermediate hosts (i.e., freshwater crabs) remains high.^4^^,^^5^^,^^15^ Consumption of reservoir hosts that feed on these infected intermediates, contamination during food preparation (i.e., hemolymph of crabs), or consumption of preserved crustacean-based products (i.e., pickled crabs and sauces) are possible reasons for how four of our patients who claimed not to consume raw crustaceans contracted paragonimiasis.^18^ This may explain the sudden increase in cases reported in 2017.

Rapid resolution was observed upon treatment with praziquantel (PZQ), with an 86% to 100% cure rate for a 75-mg/kg/day, thrice daily 2-day course, and 100% for a 3-day course.^3^^,^^19^ Dizziness, headache, and gastrointestinal distress are some possible side effects,^6^ but none were observed in this study. All patients recovered completely, but cachexic patients required longer time frames to resolve complications.^3^^,^^9^^,^^14^ This demonstrated the high efficacy of anthelmintic drugs toward treating paragonimiasis in Thai adults and children,^5^^,^^18^ with no eggs detected in 2-month follow-ups.^1^

This efficacy also highlights that the problem lies, not in treatment, but in misdiagnosis. In China, 69% to 89% of paragonimiasis cases were misdiagnosed between 2009 and 2019,^3^^,^^13^^,^^14^^,^^20^ as is often the case for early stages of infection (asymptomatic presentations or nonspecific symptoms). This delays effective treatment and increases the risk of morbidities, debilitation, and life-threatening complications.^1^^–^^3^^,^^15^ Many of our patients had their treatment delayed (1 month–1 year) as a result of misdiagnoses, allowing their condition to become severe (cases 1–3) or chronic (cases 2 and 5). Lack of awareness and access to medical care (i.e., low economic status or difficulty traveling) exacerbated this delay further.^5^ Key presentations of paragonimiasis include chronic cough with hemoptysis, fever, pleural effusion with peripheral eosinophilia, and abnormal thoracic radiographs.^3^^,^^8^^,^^12^^,^^14^^,^^17^^,^^19^ All our patients had at least one of the symptoms just listed, with the most common being abnormal thoracic findings and eosinophilia (range, 5–55%). Misdiagnosis can occur easily should deliberate tests for *Paragonimus* eggs not be performed. This is because these clinical presentations overlap with those of TB, which is also prevalent and endemic in Thailand. A presumptive diagnosis of pulmonary TB and empirical anti-TB is of general practice despite negative acid-fast bacilli sputum samples. Pleuropulmonary paragonimiasis needs to be included in clinicians’ list of differential diagnoses, particularly in endemic regions, to address underreporting, prevent misdiagnosis, and ensure rapid treatment initiation.^6^^,^^10^^,^^13^^,^^17^

Antibody detection is the most sensitive method of diagnosis, followed by egg detection in sputum then stool. However, serological methods cannot distinguish serum antibodies readily between past and present infections, which are known to persist even after anthelmintic treatments.^21^ For patients with early-onset (asymptomatic), chronic (inactive), or ectopic paragonimiasis, eggs in clinical samples may not be demonstrable.^1^^,^^2^^,^^4^^,^^5^^,^^10^^,^^13^^,^^19^ Immunodiagnostics are excellent tools to assist diagnoses.^13^^,^^19^ All patients in our study had detectable levels of egg production in sputum, stool, and pleural samples. Further laboratory analyses (see Supplemental Methods) identified *P. heterotremus* species complex (*P. heterotremus* and *P. pseudoheterotremus*) from mountain crabs collected from Loy Tong Ku, the local waterfall, as the predominant etiological agents. This supports previous literature regarding high metacercaria prevalence in intermediate hosts.

Combined educational and preventive campaigns were proposed to address sporadic and reemerging cases of paragonimiasis.^15^^,^^18^ In addition to increasing awareness in clinicians, medical outreach programs aimed toward educating local communities (particularly primary schools) would decrease cultural and habitual practices of consuming raw crustaceans and paratenic hosts.^3^^,^^4^ One example is the mass screening and prevention campaigns put forth by Miyazaki, Japan’s local government during the 1950 s and 1960 s, which reduced parasitosis prevalence significantly and prevented sporadic, reemerging cases.^6^ All patients in our study received health education to prevent reinfection. Coupling this with continuous mass treatment, sanitary improvements, and quality control of food products,^9^^,^^13^ would further regulate the number of cases in other endemic regions in Thailand (e.g., Phetchabun, Saraburi, Nakhon Nayok, Chiang Rai, Loei, Nan, Phitsanulok, and Mae Hong Son provinces).^4^^,^^5^^,^^9^^,^^11^^,^^22^

To conclude, we assessed six cases of paragonimiasis in the Umphang District to remind clinicians of this endemic, reemerging, food-born parasitosis and prevent misdiagnosis. Paragonimiasis should be included in the list of differential diagnoses to promote early treatment with PZQ, particularly for high-risk groups that habitually consume raw or undercooked intermediate or paratenic hosts, and present with chronic cough, hemoptysis, peripheral eosinophilia, and/or other thoracic radiograph abnormalities. Sputum, stool, and immunodiagnostic techniques can be coupled to confirm diagnosis.

## Supplemental files


Supplemental materials

## References

[1] Richter J, 2022. Current status of the treatment of paragonimiasis. One Health Implement Res 2: 96–107.

[2] Gong Z Miao R Shu M Zhu Y Wen Y Guo Q Liao Q Wan C, 2017. Paragonimiasis in children in southwest China: a retrospective case reports review from 2005 to 2016. Medicine (Baltimore) 96: e7265. 28640131 10.1097/MD.0000000000007265 PMC5484239

[3] Lane MA Marcos LA Onen NF Demertzis LM Hayes EV Davila SZ Nurutdinova DR Bailey TC Weil GJ, 2012. *Paragonimus kellicotti* flukes in Missouri, USA. Emerg Infect Dis 18: 1263–1267. 22840191 10.3201/eid1808.120335 PMC3414046

[4] Yoonuan T Vanvanitchai Y Dekumyoy P Komalamisra C Kojima S Waikagul J, 2008. Paragonimiasis prevalences in Saraburi Province, Thailand, measured 20 years apart. Southeast Asian J Trop Med Public Health 39: 593–600. 19058595

[5] Watthanakulpanich D Waikagul J Dekumyoy P Muangkhum P Praevanit R Mongkhonmu S, 2005. Case report: paragonimiasis in Nan Province, northern Thailand. Southeast Asian J Trop Med Public Health 36: 853–857. 16295536

[6] Nawa Y, 2000. Re-emergence of paragonimiasis. Intern Med 39: 353–354. 10830172 10.2169/internalmedicine.39.353

[7] Doanh PN Horii Y Nawa Y, 2013. *Paragonimus* and paragonimiasis in Vietnam: an update. Korean J Parasitol 51: 621–627. 24516264 10.3347/kjp.2013.51.6.621 PMC3916448

[8] Petborom P Linasmita P Kulpraneet M, 2016. Coinfection of pulmonary paragonimiasis and pulmonary tuberculosis in Thailand. J Med Assoc Thai 99: S231–S236. 29906052

[9] Waree P Polseela P Pannarunothai S Pipitgool V, 2001. The present situation of paragonimiasis in endemic area in Phitsanulok Province. Southeast Asian J Trop Med Public Health 32: 51–54. 12041605

[10] Shim Y-S Cho S-Y Han Y-C, 1991. Pulmonary paragonimiasis: a Korean perspective. Seminars in Respiratory Medicine. Thieme Medical Publishers, 35–45.

[11] Miyazaki I Harinasuta T, 1966. The first case of human paragonimiasis caused by *Paragonimus heterotremus* Chen et Hsia, 1964. Ann Trop Med Parasitol 60: 509–514.

[12] Kanpittaya J Sawanyawisuth K Vannavong A Intapan PM Maleewong W Zhang W Strobel M, 2010. Different chest radiographic findings of pulmonary paragonimiasis in two endemic countries. Am J Trop Med Hyg 83: 924–926. 20889893 10.4269/ajtmh.2010.10-0091 PMC2946770

[13] Blair D, 2022. Lung flukes of the genus *Paragonimus*: ancient and re-emerging pathogens. Parasitology 149: 1286–1295. 35292126 10.1017/S0031182022000300 PMC10090773

[14] Lane MA Barsanti MC Santos CA Yeung M Lubner SJ Weil GJ, 2009. Human paragonimiasis in North America following ingestion of raw crayfish. Clin Infect Dis 49: e55–e61. 19681705 10.1086/605534

[15] Sohn BS Bae Y-J Cho YS Moon H-B Kim T-B, 2009. Three cases of paragonimiasis in a family. Korean J Parasitol 47: 281–285. 19724703 10.3347/kjp.2009.47.3.281 PMC2735695

[16] Saborio P Lanzas R Arrieta G Arguedas A, 1995. *Paragonimus mexicanus* pericarditis: report of two cases and review of the literature. J Trop Med Hyg 98: 316–318. 7563258

[17] Hwang K-E Song H-Y Jung J-W Oh S-J Yoon K-H Park D-S Jeong E-T Kim H-R, 2015. Pleural fluid characteristics of pleuropulmonary paragonimiasis masquerading as pleural tuberculosis. Korean J Intern Med 30: 56–61. 25589836 10.3904/kjim.2015.30.1.56 PMC4293564

[18] Robinson MW Sotillo J, 2022. Foodborne trematodes: old foes, new kids on the block and research perspectives for control and understanding host–parasite interactions. Parasitology 149: 1257–1261. 35734871 10.1017/S0031182022000877 PMC11010571

[19] Yoshida A Doanh PN Maruyama H, 2019. *Paragonimus* and paragonimiasis in Asia: an update. Acta Trop 199: 105074. 31295431 10.1016/j.actatropica.2019.105074

[20] Im J-G Whang H Kim W Han M Shim Y Cho S-Y, 1992. Pleuropulmonary paragonimiasis: radiologic findings in 71 patients. AJR Am J Roentgenol 159: 39–43. 1609718 10.2214/ajr.159.1.1609718

[21] Maleewong W Wongkham C Intapan P Pariyanonda S Morakote N, 1992. Excretory-secretory antigenic components of *Paragonimus heterotremus* recognized by infected human sera. J Clin Microbiol 30: 2077–2079. 1500515 10.1128/jcm.30.8.2077-2079.1992 PMC265445

[22] Ekarohit D Chesdapan C Thitasut P Sukonthasan K Choochote W, 1991. Paragonimiasis in Mae Hong Son Province northern Thailand: case report. Southeast Asian J Trop Med Public Health 22: 340–341. 1822921

