# Supplementary material for: Case Report: Reemerging Paragonimiasis in Umphang District, Thailand
Source: Am J Trop Med Hyg. 2023 Feb 27;108(4):738–43. doi: 10.4269/ajtmh.22-0708 (PMC10077016; doi:10.4269/ajtmh.22-0708)
Supplement: Supplementary file 1 [file tpmd220708.SD1.pdf]

Supplementary Methods

Study Design

An outbreak of six cases of paragonimiasis were identified in 2017 within the Karan hill-tribe near the Thai-Myanmar border in Umphang district, Tak province, Western Thailand. Extensive medical histories were performed at Umphang Community Hospital for all patients before treatment with 75-80 mg/kg/day (MKD) praziquantel (PZQ), fractionated thrice-daily (TID) for 2-5 days (depending on severity of infection). Other treatments and therapies were administered as necessary for each case. Thoracic radiographs were compared before and after treatment (during 2-7-month follow-ups) to determine the efficacy of treatment. Race was not included as a direct predisposing factor. The cultural practice of consuming raw, undercooked, or preserved crustacean-based products was inquired as a predisposing factor for *Paragonimus* spp. infestation.

Crab Preparation

The gills, hearts, viscera, and muscle of the crabs were compressed between large glass plates and separately examined under a stereomicroscope. The legs were ground in physiological saline using a blender. The mixture was transferred into a sedimentation flask and set to stand for 30 minutes. This stage was repeated until the supernatant became clear. The sediment was then examined for metacercaria under a stereomicroscope.

DNA Extraction, Primer Design, and PCR

DNA extraction was performed on the visceral organs of the mountain crabs as well as sputum samples of the 3- and 10-year-old Karen boys using FavorPrep™ Tissue Genomic DNA

Extraction Mini Kit (Favorgen® Biotech Corp.). PCR specific primers (*Pps*COI) were designed for the *Cox1* gene of *P. pseudoheterotremus* (accession number: EF446317) through the National Center for Biotechnology Information (NCBI) and sequenced accordingly: forward primer 5'-ACATATTTGCATGACTCTAACTAAC-3' and reverse primer 5'-GAACCAAGGGTCATGCAGCAA-3'. Gene amplification was performed using a Mastercycler® PCR Thermal Cycler (Eppendorf™, Germany) set to the following conditions: 94°C for 5 min; 35 cycles at 94°C for 1 min, 45°C for 30 s, and 72°C for 1 min; and 72°C for 5 min.

Supplementary Results

Case 1: 3-year-old boy

A right thoracentesis was performed and revealed straw-colored pleural fluid. Microscopic examination found a RBC of 3,200 cell/mm^3 and WBC of 4,320 cell/mm^3 (69% polymorphonuclear leukocytes, 14% lymphocytes, and 17% eosinophils). Chemistry results revealed a pH of 9.0, glucose <5 mg/dL, protein 7.1 g/dL, and ADA 45.3 U/L. Microbiological examination through fresh smears revealed seven *Paragonimus* eggs per slide (Supplementary Figure 2). Stool examination also revealed *Paragonimus* eggs.

Case 3: 10 year old boy

Thoracocentesis was performed and revealed yellow-colored fluid. Microscopic examination found an RBC of 270 cell/mm^3, WBC 450 cell/mm^3 (33% polymorphonuclear leukocytes, 21% lymphocyte, and 46% monocyte). Chemistry results revealed a glucose of 0 mg/dL, protein 9.1

g/dL (serum protein 8.7 g/dL), fluid LDH 1,814 U/L (serum LDH 266 U/L), and ADA 41.6 U/L. Microbiological examination found cholesterol crystals in fresh sputum smear.

He was moved to a tertiary hospital in Phitsanulok province. Thoracentesis was repeated and microscopic examination showed RBC 4,000 cell/mm^3 and WBC 3,940 cell/mm^3 (12% polymorphonuclear leukocytes, 69% lymphocyte, and 8% eosinophil). Chemistry test results found a pH of 7.46, triglyceride 19.1 mg/dL, cholesterol 91 mg/dL, glucose 0 mg/dL, protein 8 g/dL, LDH 11.9 U/L.

Supplementary Figures

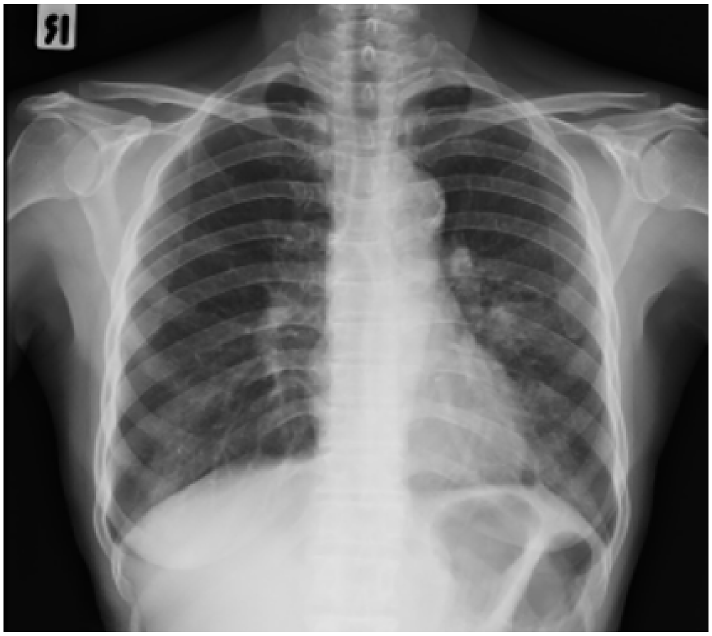

Supplementary Figure 1. Thoracic radiograph of a 55-year-old female 1 year before visiting Umphang's community hospital.

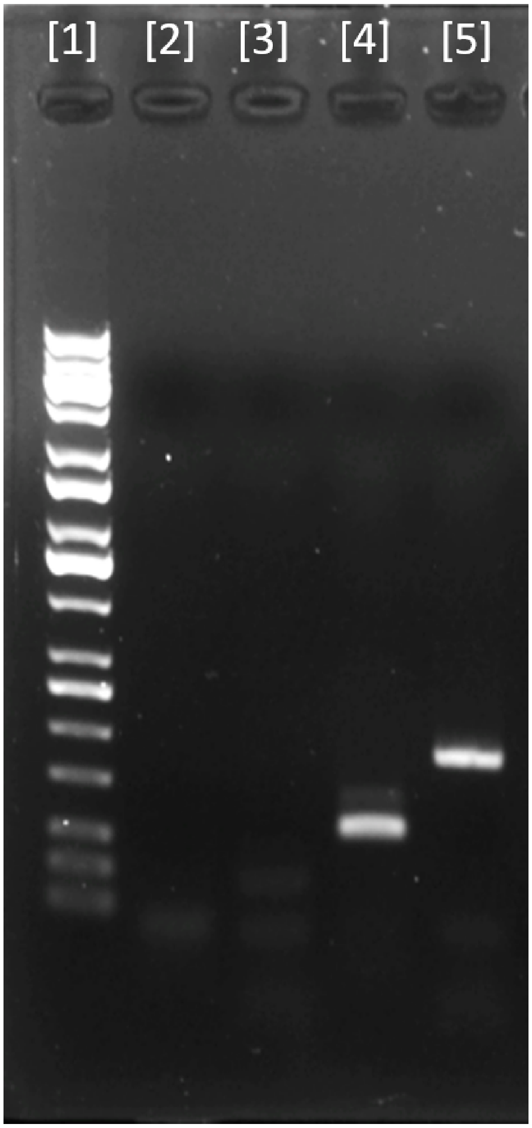

Supplementary Figure 2. Agarose gel electrophoresis results of Cox1 PCR products. [1] 100 bp ladder, [2] negative sputum result of 3-year-old Karen boy (Case 1), [3] negative sputum result of 10-year-old Karen boy (Case 3), [4] *P. heterotremus* metacercaria (n = 6), and [5] *P. pseudoheterotremus* metacercaria (n = 6).

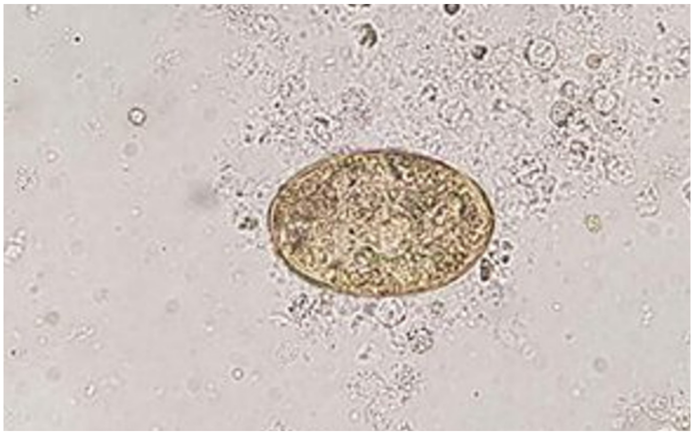

Supplementary Figure 3. Paragonimus egg found in pleural effusion of 3-year-old Karen male (Case 1).
